# The complete chloroplast genome sequence of *Rubus eucalyptus* (Rosaceae)

**DOI:** 10.1080/23802359.2019.1664346

**Published:** 2019-09-12

**Authors:** Qiang Zhu, Ying Tian, Wenyu Liang

**Affiliations:** a The State Key Laboratory of Seeding Bioengineering, Ningxia Forestry Institute Yinchuan, Yinchuan, China;; b School of Agriculture, Ningxia University, Yinchuan, China;; c School of Life Sciences, Ningxia University, Yinchuan, China

**Keywords:** Rubus eucalyptus, Rosaceae, chloroplast genome, phylogenetic tree

## Abstract

*Rubus eucalyptus* Focke belong to genus *Rubus* in the family Rosaceae, the fresh fruits of which can be used for medicine and cosmetic. The plastome of *R. eucalyptus* is 155,672 bp in length, with one large single copy region of 85,277 bp, one small single copy region of 18,864 bp, and two inverted repeat (IR) regions of 25,748 bp. It contains 130 genes, including 86 protein-coding genes, 8 ribosomal RNA, and 37 transfer RNA. Phylogenetic tree shows that *R. eucalyptus* was at the basal of Rosaceae. The published plastome within *Rubus* provides significant insight for elucidating the phylogenetic relationship of taxa within tribe Rosaceae.

*Rubus eucalyptus* Focke belong to genus *Rubus* in the family Rosaceae, distributed in Guizhou, Sichuan, Shanxi, Hubei and Yunnan provinces in China (Robertson 1974; Thompson 1995). The fresh Fruits can be used for medicine and cosmetic. Because of the complexity of their inter- and intraspecific morphological variations, species delimitation in genus *Rubus* is quite difficult (Alice and Campbell 1999). A well-resolved phylogeny based on sufficient molecular markers is essential to understanding the relationship between species, and the efficient utilization and improvement of these wild *Rubus* species as crop. In this study, we reported the complete chloroplast genome of *R. eucalyptus*, a wild species widespread in subtropical and tropical China, as a resource for future studies on the taxonomy of *Rubus*.

Fresh leaves of *R. eucalyptus* were collected from Xiaozhaizigou National Reserve (Mianyang, Sichuan, China；coordinates: 103°45′E, 31°50′N). Dried and kept in silica gel for DNA extraction, and then stored in the Herbarium of Key laboratory in Ningxia Forestry Institute with the accession number of NX190512. Total genomic DNA was extracted with a modified CTAB method (Doyle and Doyle 1987). First, we obtained 10 million high quality pair-end reads for *R. eucalyptus*, and after removing the adapters, the remained reads were used to assemble the complete chloroplast genome by NOVOPlasty (Dierckxsens et al. 2017). The complete chloroplasts genome sequence of *Rubus coreanus* was used as a reference. Plann v1.1 (Huang and Cronk 2015) and Geneious v11.0.3 (Kearse et al. 2012) were used to annotate the chloroplast genome and correct the annotation. The completecp genome sequence has been submitted to GenBank (accession number MN013402).

The *R. eucalyptus* cp genome is 155,672 bp in length, exhibits a typical quadripartite structural organization, consisting of a large single copy (LSC) region of 85,277 bp, two inverted repeat (IR) regions of 25,796 bp and a small single copy (SSC) region of 18,864bp. The cp genome contains 130 complete genes, including 86 protein-coding genes (86 PCGs), eight ribosomal RNA genes (four rRNAs), and 37 tRNA genes (20 tRNAs). Most genes occur in a single copy, while 15 genes occur in double, including all rRNAs (4.5S, 5S, 16S, and 23S rRNA), 5 tRNAs (trnA-UGC, trnL-CAA, trnN-GUU, trnR-ACG, and trnV-GAC), and 6 PCGs (rps7, rps12, rpl2, rpl23, ndhB, ycf2). The overall AT content of cp DNA is 62.9%, while the corresponding values of the LSC, SSC, and IR regions are 65.1%, 69.1%, and 57.2% respcetively.

In order to further clarify the phylogenetic position of *Rubus eucalyptus*, plastome of six representative Rosaceae species were obtained from NCBI to construct the plastome phylogeny, with *Malus transitoria* as an outgroup. All the sequences were aligned using MAFFT v.7.313 (Katoh and Standley 2013) and maximum likelihood phylogenetic analyses were conducted using RAxML v.8.2.11 (Stamatakis 2014). The phylogenetic tree shows that all species of Rosaceae were clustered together one clade. while *R. eucalyptus* was at the basal of Rosaceae (Figure 1). This study provides a platform for future molecular phylogenetics of species in tribe Rosaceae.

**Figure 1.**
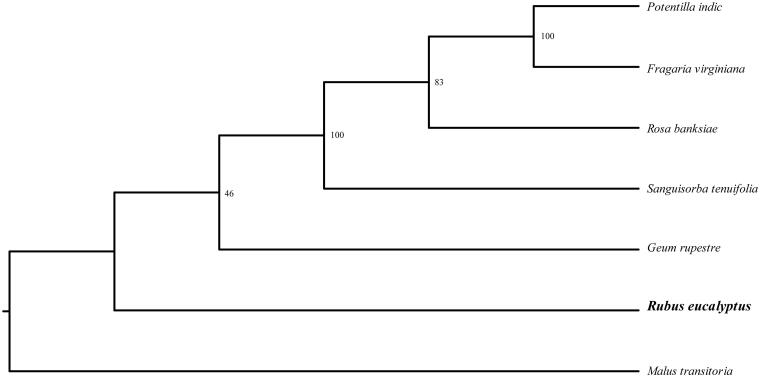
Phylogenetic relationships of Rosaceae species using whole chloroplast genome. GenBank accession numbers: *Fragaria virginiana* (KY085911.1), *Geum rupestre* (NC_037392.1), *Malus transitoria* (MK098838.1), *Potentilla indic* (NC_041178.1), *Rosa banksiae* (NC_042194.1), *Sanguisorba tenuifolia* (NC_042223.1).
